# Long-term oral administration of a novel estrogen receptor beta agonist enhances memory and alleviates drug-induced vasodilation in young ovariectomized mice

**DOI:** 10.1016/j.yhbeh.2021.104948

**Published:** 2021-03-08

**Authors:** Aaron W. Fleischer, Jayson C. Schalk, Edward A. Wetzel, Alicia M. Hanson, Daniel S. Sem, William A. Donaldson, Karyn M. Frick

**Affiliations:** a Department of Psychology, University of Wisconsin-Milwaukee, Milwaukee, WI 53211, United States of America; b Department of Chemistry, Marquette University, Milwaukee, WI 53201-1881, United States of America; c Department Pharmaceutical Sciences, Concordia University Wisconsin, Mequon, WI 53097, United States of America; d Center for Structure-Based Drug Design and Development, Concordia University Wisconsin, Mequon, WI 53097, United States of America

**Keywords:** ERβ, Menopause, Ovariectomy, Anxiety, Depression, Object placement, Object recognition, Hot flash, Mouse

## Abstract

Development of estrogen therapies targeting the β (ERβ) but not α (ERα) estrogen receptor is critically needed for the treatment of negative menopausal symptoms, as ERα activation increases health risks like cancer. Here, we determined the effects of long-term oral treatment with EGX358, a novel highly selective ERβ agonist, on memory, vasodilation, and affect in young ovariectomized mice. Mice were orally gavaged daily for 9 weeks with vehicle, 17β-estradiol (E_2_), the ERβ agonist diarylpropionitrile (DPN), or EGX358 at doses that enhance memory when delivered acutely. Tail skin temperature was recorded as a proxy for vasodilation following injection of vehicle or senktide, a tachykinin receptor 3 agonist used to model hot flashes. Anxiety-like behavior was assessed in the open field (OF) and elevated plus maze (EPM), and depression-like behavior was measured in the tail suspension (TST) and forced swim tests (FST). Finally, memory was assessed in object recognition (OR) and object placement (OP) tasks. E_2_, DPN, and EGX358 reduced senktide-mediated increases in tail skin temperature compared to vehicle. All three treatments also enhanced memory in the OR and OP tasks, whereas vehicle did not. Although E_2_ increased time spent in the center of the OF, no other treatment effects were observed in the OF, EPM, TST, or FST. These data suggest that long-term ERβ activation can reduce hot flash-like symptoms and enhance spatial and object recognition memories in ovariectomized mice. Thus, the highly selective ERβ agonist EGX358 may be a promising avenue for reducing menopause-related hot flashes and memory dysfunction.

## 1. Introduction

The menopausal loss of circulating estrogens is often associated with many negative symptoms. Among these, hot flashes, memory loss, affective symptoms, and weight gain are the most frequently reported (Bosworth et al., 2001; Bromberger et al., 2013; Epperson et al., 2013; Lovejoy, 1998; Mitchell and Woods, 2001; Woods and Mitchell, 2005). In addition to their individual impacts on women’s quality of life, some of these negative symptoms synergistically disrupt menopausal women’s lifestyles. For example, menopause-related depression, anxiety, sleep disturbances, and objective hot flashes all impede memory (Drogos et al., 2013; Greendale et al., 2010; Maki et al., 2008, 2020), and menopausal weight gain is positively correlated with hot flash frequency and negatively correlated with self-esteem (Gold et al., 2000). Although estrogen-based hormone therapies (HTs) are often effective in reducing many of these negative symptoms when initiated early in the menopausal transition (Maki, 2013), HT is also associated with increased risks of breast and uterine cancer, among other health issues (Rossouw et al., 2002). More recent clinical and preclinical findings have demonstrated that the negative side effects of HTs may be due to activation of the alpha (ERα), but not beta (ERβ), estrogen receptor isoform (Alevizaki et al., 2007; Lin et al., 2015; Péqueux et al., 2012; Shearman et al., 2003, 2005). Therefore, future estrogen-based HTs should selectively target ERβ to avoid the health risks associated with ERα activation.

There are several ways to target ERβ selectively to treat menopausal symptoms. The most common form of ERβ-selective treatment is through the consumption of phytoestrogens. Relative to placebo, daily phytoestrogen administration in menopausal women reduces hot flash incidence and severity (Cheng et al., 2007; Grady et al., 2009), reduces anxiety and depression (Casini et al., 2006; Davinelli et al., 2017), and improves memory in digit symbol and digit span tests (Casini et al., 2006). However, phytoestrogens comprise a class of many different plant-based compounds, making it difficult to parse the effects of any given compound on symptoms. Additionally, although phytoestrogens more selectively activate ERβ than ERα, their selectivity is generally low, with the most common class, isoflavones, demonstrating a range between 8-and 68-fold selectivity (Cheng et al., 2007; Nikov et al., 2000). Synthetic agonists, such as LY500307, WAY200070, and diarylpropionitrile (DPN) also show relatively low selectivity for ERβ, namely 14-, 68-, and 70-fold, respectively (Hanson et al., 2018; Malamas et al., 2004; Meyers et al., 2001; Norman et al., 2006). Among these, DPN is often used in preclinical studies. However, the concentration of DPN that selectively activates ERβ transcriptional activation without concomitant ERα transcriptional activation is only 45% the extent of E_2_, suggesting a need for more selective ERβ agonists (Meyers et al., 2001)..

We recently synthesized a novel ERβ agonist, 4-[trans-(4-hydroxymethyl)cyclohexyl]phenol (EGX358; formerly called ISP358–2), which has a roughly 750-fold selectivity for ERβ over ERα, and is, therefore, the most selective synthetic ERβ agonist to date (Hanson et al., 2018). Additionally, immediate acute post-training administration of EGX358 enhanced memory consolidation in object placement (OP) and object recognition (OR) tasks of spatial and object identity memory, respectively, when administered via oral gavage, intraperitoneal injection, or direct bilateral infusion into the dorsal hippocampi of young bilaterally ovariectomized (OVX) female mice (Hanson et al., 2018). Although these data suggest promising efficacy for memory loss, we have not yet tested the effects of long-term administration of this compound on memory or other preclinical indices of menopausal symptoms, which is critical for its development as a potential new HT option.

Bilateral OVX of young female rodents is a commonly used model to study ovarian hormone loss. Although the rapid cessation of ovarian hormone synthesis produced by surgical removal of the ovaries in young females does not recapitulate the gradual hormone decline that results from reproductive senescence in women, long-term OVX in rats and mice is associated with common menopause-like outcomes such as weight gain (Couse and Korach, 1999; Sibonga et al., 1998), anxiety-and depression-like symptoms (Imwalle et al., 2005; Lund et al., 2005; Rocha et al., 2005; Walf et al., 2004; Walf and Frye, 2005a, 2006), and impaired hippocampus-dependent spatial long-term and working memory, as well as object recognition memory (Heikkinen et al., 2002, 2004; Singh et al., 1994; Wallace et al., 2006). Moreover, ERβ activation reduces weight gain induced by high-fat diets (Foryst-Ludwig et al., 2008), enhances hippocampal memory (Boulware et al., 2013; Jacome et al., 2010; Pereira et al., 2014; Walf et al., 2008), and reduces anxiety-and depression-like behaviors in OVX rodents (Lagunas et al., 2010; Li et al., 2014; Lund et al., 2005; Oyola et al., 2012; Walf and Frye, 2005a, 2007). Hot flashes, sudden sensations of heat accompanied by vasodilation and a heat dissipation response (e.g., sweating), are not known to be increased by OVX in rodents, but indices of vasodilation are influenced by OVX and drug treatments. Vasodilation can be measured in rats and mice by assessing tail skin temperature (T_Skin_), which rises to release excess heat. OVX gradually increases T_Skin_ in both rats and mice, although this process often takes weeks (Opas et al., 2004, 2006; Zhao et al., 2011), which does not suitably recapitulate transient hot flashes experienced during menopause. Administration of the tachykinin 3 receptor agonist, senktide, induces rapid and transient neurokinin B-mediated vasodilation, and has recently been used to study the neural mechanisms underlying hot flashes (Krajewski-Hall et al., 2018; Krull et al., 2017; Padilla et al., 2018). However, the effects of ERβ signaling in this model are currently unknown. Collectively, despite the shortcomings of OVX for modeling reproductive senescence, data support the use of OVX and senktide as appropriate first steps in which to begin testing the potential for EGX358 to relieve menopausal symptoms.

Development of ERβ-selective HT methods is needed, as current treatment options have relatively low selectivity and may thus inadvertently promote the ERα-mediated side effects of current HTs. Given EGX358’s high selectivity for ERβ, it is important to determine whether this compound provides benefits similar to commercially available ERβ agonists. If so, EGX358 could be further developed as a therapeutic agent for alleviating menopause-related symptoms with minimal off-target ERα activation. Although we have demonstrated previously that acute EGX358 treatment promotes memory consolidation in OVX mice without facilitating breast cancer cell proliferation, binding other nuclear hormone receptors, or influencing blood chemistry or organ tissue morphology (Hanson et al., 2018), we have not previously examined the effects of its long-term administration on preclinical indices of menopausal symptoms. Therefore, the present study measured the effects of long-term EGX358 treatment via daily oral gavage on memory, senktide-induced vasodilation, anxiety-and depression-like behaviors, and body weight in OVX mice. Our findings suggest that long-term treatment with EGX358 prevents senktide-induced vasodilation and enhances spatial and object recognition memory to similar extents as the potent estrogen 17β-estradiol (E_2_) and commercially available ERβ agonist DPN, but does not affect anxiety-or depression-like behaviors or body weight.

## 2. Methods

### 2.1. Subjects

As an initial step to test the efficacy of EGX358 in reducing preclinical indices of menopause-related symptoms, all subjects were eight-week-old female C57BL/6 mice (*n* = 40), obtained from Taconic Biosciences (Germantown, New York). Mice were housed five per cage until 24 h prior to surgery. Following surgery, mice were singly housed for the duration of the experiment. Mice were maintained on a 12-hour light-dark cycle (lights on at 07:00) and were given food and water access ad libitum. Diet was standard rodent chow (Teklad Rodent Diet 8604, Envigo, Madison, WI), which contained isoflavone equivalents of daidzein and genistein aglycone ranging from 350 to 650 mg/kg. Handling to acclimate mice to the experimenters began one week after surgery. All treatments and behavioral testing occurred during light hours. All procedures followed the National Institutes of Health Guide for the Care and Use of Laboratory Animals and were approved by the University of Wisconsin-Milwaukee Institutional Animal Care and Use Committee.

### 2.2. General experimental design

**Fig. 1.**
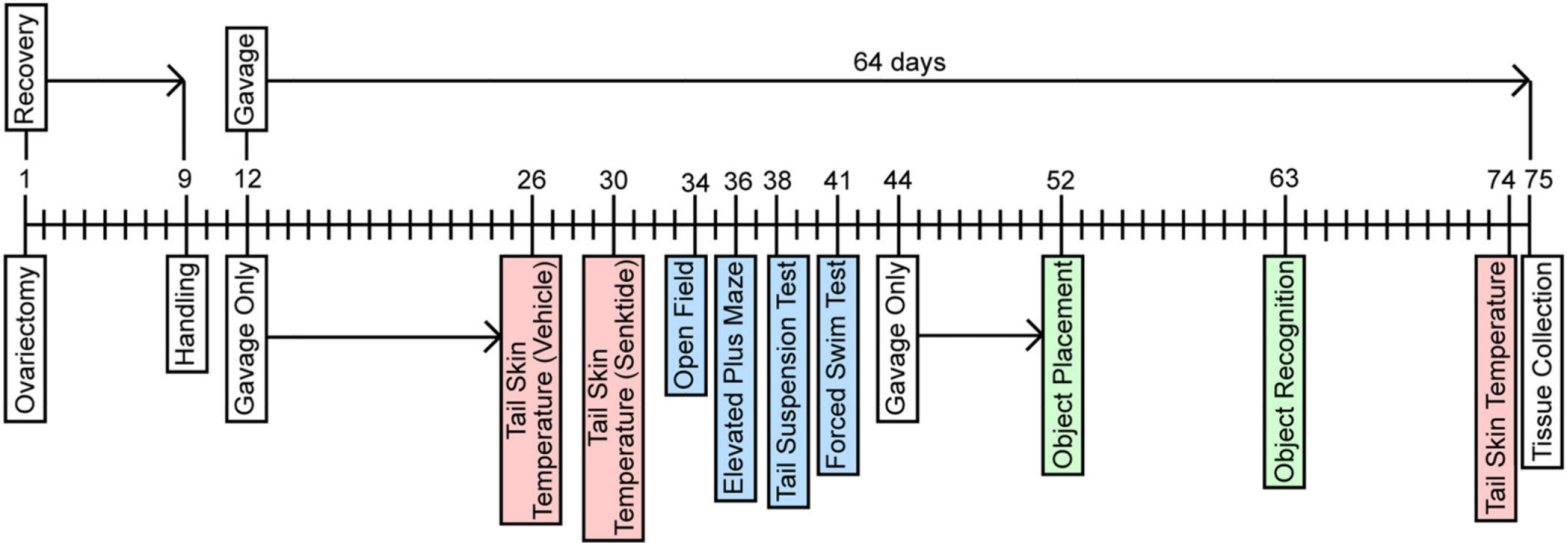
Experimental timeline. Young female C57BL/6 mice were bilaterally ovariectomized at approximately 8 weeks of age. Mice recovered for 7–8 days, after which wound clips were removed. Mice were handled for 30 s/day on days 9–11, and on day 12, mice began daily oral gavage treatment (10% DMSO, E_2_, DPN, or EGX358), which continued for the duration of the experiment, for 64 days in total. During the first 2 weeks of treatment, mice were also subcutaneously injected with 0.9% saline every other day. Following the first 2 weeks of treatment, mice began testing, which was conducted in the following order: T_Skin_ with subcutaneous injection of 0.9% saline (vehicle), T_Skin_ with subcutaneous injection of senktide, open field, elevated plus maze, tail suspension test, forced swim test. After completing the forced swim test, mice were gavaged without further testing for 8 days. On day 52, mice began object placement and, subsequently, object recognition training and testing. On day 74, mice were thermally imaged for T_Skin_ 1 h following gavage. Finally, on day 75 of the experiment, mice were gavaged and euthanized 30 min later, and brain tissue was rapidly dissected and stored at −80 °C. Mice were gavaged 1 h prior to each behavioral test. DMSO, dimethyl sulfoxide; E_2_, 17β-estradiol; DPN, diarylpropionitrile; T_Skin_, tail skin temperature.

**Fig. 2.**
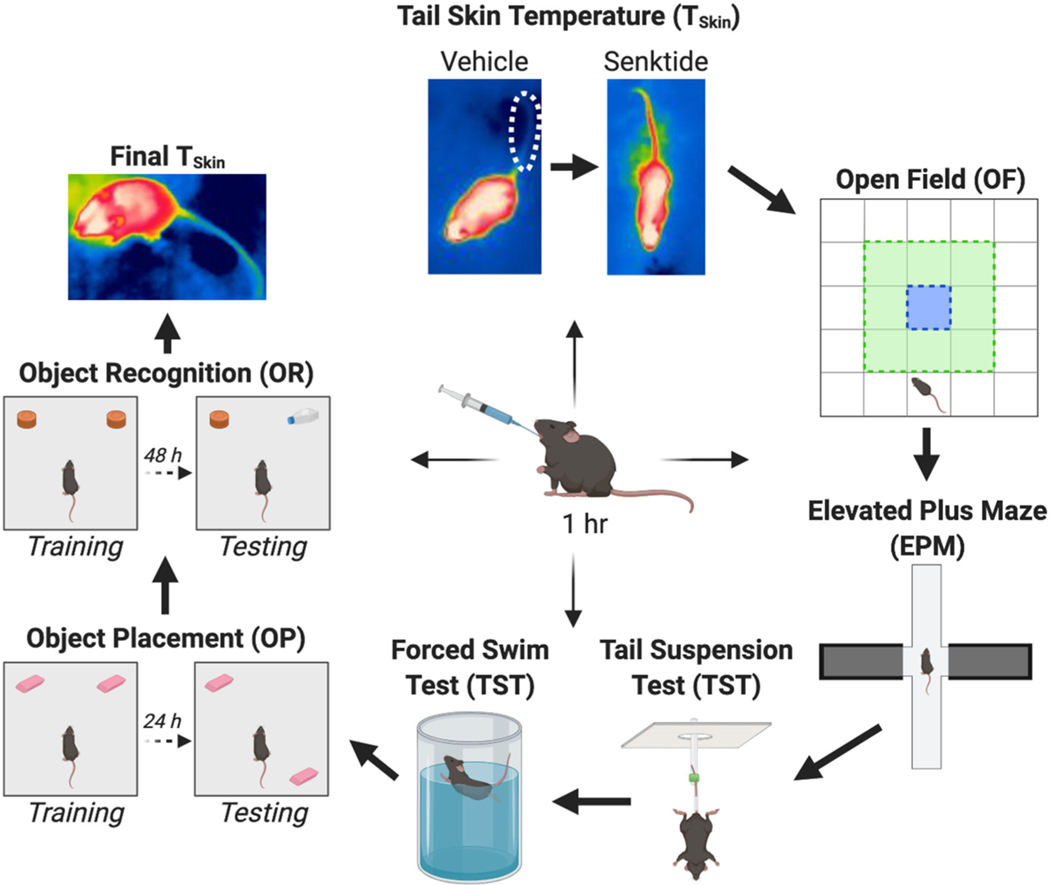
Visual representations of tasks conducted. Young, ovariectomized mice were gavaged with 10% DMSO, E_2_, DPN, or EGX358 1 h prior to each behavioral or physiological measurement. Diagrams represent each behavioral apparatus. Mice were thermally imaged for tail skin temperature following subcutaneous injection of 0.9% saline and, subsequently, senktide for 30 min. Next, mice were measured for anxiety-like and locomotor behaviors in the open field, namely time spent in and entries into each zone, as well as distance traveled during the 10-min test. Mice were then measured for anxiety-like behaviors in the elevated plus maze for 10 min, quantified as entries into and time spent in each set of arms and the center space. Mice were measured for time spent immobile and latency to first bout of immobility as proxies of depression-like behaviors in the tail suspension test for 6 min and, subsequently, the forced swim test for 4 min. Mice were tested for memory consolidation in the object placement and object recognition tasks by measuring time spent with the moved or novel objects, respectively, during the testing phase of each task. Finally, mice were thermally imaged for tail skin temperature 1 h after gavage on the final day of the experiment. DMSO, dimethyl sulfoxide; E_2_, 17β-estradiol; DPN, diarylpropionitrile. Created with BioRender.com.

The experimental timeline for the study is illustrated in Fig. 1, with additional visual representations of the procedures used depicted in Fig. 2. Three days after arrival in the lab, mice were ovariectomized (Fig. 1). Mice were weighed immediately prior to surgery and once weekly throughout the remainder of the study to appropriately dose treatments and to test for effects of treatment on weight gain. Following recovery from OVX surgery and three days of handling, mice were gavaged daily with their respective treatments for two weeks in their colony room without being tested. During this two-week period, mice were also given subcutaneous (SC) injections of 0.9% saline every other day to acclimate them to injection stress prior to vasodilation measurements. Daily gavage continued throughout the duration of the study, and mice were gavaged in their colony room 1 h prior to testing on days of behavioral and physiological measurements, unless otherwise noted. This timepoint was chosen because of the number and variety of tasks in the current study. Due to the wide variety of times at which estrogenic compounds elicit behavioral (Bekku and Yoshimura, 2005; Bernardi et al., 1989; Estrada-Camarena et al., 2003; Gresack and Frick, 2006b; Harburger et al., 2007; Li et al., 2014; Lund et al., 2005; Oyola et al., 2012) and neural effects (Eid et al., 2020; Galea, 2008; Inagaki et al., 2010, 2012; Jacome et al., 2010; Phan et al., 2012) when administered peripherally to ovariectomized rats and mice, and given that the time frame in which systemically-administered EGX358 activates ERβ in the brain is currently unknown, 1 h was chosen to allow sufficient time for the compound to cross the blood-brain barrier and bind ERβ in target brain regions. Mice were first tested for vasodilation responses to vehicle, and then senktide, administration. Next, anxiety-like behaviors were measured in the open field (OF) and elevated plus maze (EPM). Then, depression-like behaviors were measured in the tail suspension test (TST) and forced swim test (FST). Following completion of FST, mice did not undergo any testing for one week to mitigate potential effects of accumulated stress on memory tests. Then, mice were trained and tested in OP and OR to assess memory. Finally, mice were thermally imaged one day following memory testing to determine long-term effects of treatment on baseline thermal regulation. Following completion of all measurements, mice were gavaged and euthanized 30 min later, and tissue from ERβ-laden brain regions implicated in menopause-related symptoms was rapidly dissected and collected on wet ice and frozen at −80 °C for follow-up biochemical studies at a later date.

All behavioral tasks were conducted in lighting <100 lx and ambient room temperature of 23 ± 2 °C. To reduce the chances of mice associating a given behavioral testing room with stressful situations, tests were conducted in three separate rooms: vehicle and senktide vasodilation measures, TST, and FST were conducted in one; OF, OP, and OR were conducted in a second; and EPM was conducted in the third. Furthermore, all gavage procedures and final thermal images were conducted in the colony room. Additionally, during all behavioral and physiological testing, a researcher blind to treatment initiated recordings in rooms adjacent to those in which the behavior was being conducted, and researchers remained outside of the testing rooms during testing. All behavioral and physiological measurements were quantified and recorded by researchers blind to treatment following completion of a given task, unless otherwise noted.

### 2.3. Ovariectomy surgery

Mice were singly housed 24 h prior to surgery. On the day of surgery, mice were bilaterally ovariectomized using a procedure that our laboratory has repeatedly employed to eliminate circulating estrogens (Frick and Berger-Sweeney, 2001; Gresack and Frick, 2006a; Hanson et al., 2018; Koss et al., 2018; Tuscher et al., 2016a), as previously verified by vaginal lavage (Frick and Berger-Sweeney, 2001). Briefly, on the day of surgery, mice were anesthetized with isoflurane (5% for induction, 2% for maintenance). Immediately after anesthesia induction, mice were given a SC injection of Rimadyl (1:100, 10 mL/kg) for analgesia. Mice were then shaved bilaterally on their flanks and incised, after which their ovaries, oviducts, and tips of the uterine horns were ligated with monofilament and then removed, as confirmed by visual observation. Muscle wall incisions were sutured with chromic gut, and skin incisions were closed with wound clips. Mice recovered for seven days, the first two days of which they were given SC Rimadyl injections for post-operative analgesia. Following this recovery period, wound clips were removed, and mice were replaced in their colony room.

### 2.4. Drugs and administration

Following recovery from surgery and wound clip removal, mice were handled briefly (30 s/day) for three days to habituate to experimenter handling (Fig. 1). The next day, mice began daily gavage treatment (*n* = 10/group) in the colony room using bulb-tipped gastric gavage needles (24 GA, 25 mm) (Hanson et al., 2018). Mice were gavaged daily for two weeks prior to the start of testing and throughout the rest of the study. On days of training or testing, mice were gavaged 1 h prior to testing (Fig. 2). Mice received β-cyclodextrin-encapsulated E_2_ (Sigma-Aldrich, 0.2 mg/kg), DPN (Tocris Biosciences, 0.05 mg/kg), or EGX358 (0.5 mg/kg), at doses previously shown to enhance hippocampus-dependent memory consolidation when administered systemically (Gresack and Frick, 2006a; Hanson et al., 2018). Given the hydrophobicity of EGX358 (Hanson et al., 2018), we have previously dissolved this compound in 10% DMSO when acutely treating mice peripherally via gavage or intraperitoneal injection (Hanson et al., 2018). Thus, each treatment in the current study was dissolved in 10% DMSO in 0.9% saline, and all treatments were administered at 10 mL/kg, as described previously (Hanson et al., 2018). Vehicle-treated mice received 10% DMSO in 0.9% saline daily. To acclimate the mice to injection stress prior to vasodilation measurements, mice were given a SC injection of 0.9% saline (5 mL/kg) every other day during the two weeks prior to behavioral testing. During vasodilation experiments, mice were given a single SC injection of either vehicle (0.9% saline, 5 mL/kg) or senktide (Tocris Biosciences, 0.5 mg/kg, 5 mL/kg) dissolved in 0.9% saline.

### 2.5. Tail vasodilation response

To measure the effects of long-term treatment on vasomotor symptoms, tail skin temperature (T_Skin_), a proxy for vasodilation (Fig. 2), was measured three times, as described previously (Krajewski-Hall et al., 2018; Krull et al., 2017; Padilla et al., 2018). On days of vehicle or senktide challenge, each mouse was transported to a testing room immediately following gavage and given 1 h to acclimate to the new environment prior to testing. In its home cage, each mouse was placed in a secondary container (10 in × 18 in x10 in) beneath a thermal camera (E8, FLIR, Wilsonville, OR, USA). After acclimation, the cage top was removed and a researcher in an adjacent room initiated continual thermal imaging (FLIR Tools+). Baseline T_Skin_ was recorded for 10 min to ensure there were no effects of stress or activity due to removal of the cage top. Then, a researcher removed the mouse from its home cage and injected it SC with vehicle or senktide. The mouse was then returned to its home cage, and T_Skin_ was recorded for an additional 20 min. After testing, the cage top was placed back onto the cage, and the mouse was removed from the testing room. On the final day of the study, mice were imaged once in their colony room 1 h following gavage to determine the effects of long-term treatment with E_2_, DPN, or EGX358 on T_Skin_ following ovariectomy.

Following completion of vasodilation measurements, T_Skin_ from these experiments was quantified using FLIR Tools+ software. T_Skin_ was measured by averaging the temperature of the tail in a 1-cm line beginning 2 cm from the tail base, as described by others (Krull et al., 2017). During the vehicle and senktide challenges, the researcher quantified T_Skin_ immediately following the removal of the cage top, as well as at 5 and 7.5 min following cage top removal to ensure that temperature changes were not due simply to stress or increased activity associated with the removal of the cage top. Beginning immediately following SC injection, T_Skin_ was measured every minute for 20 min to assess the acute effects of vehicle or senktide administration. When a tail was obscured at the exact minute of the recording, T_Skin_ was measured at the next full second when the tail was exposed. Mice were analyzed for change in T_Skin_ (ΔT_Skin_) due to injection, which was calculated via the following equation: (TSkin, Raw – TSkin, Baseline), where TSkin, Raw was the TSkin quantification at a given time point, and TSkin, Baseline was the TSkin at 7.5 min following cage top removal, such that changes in temperature were normalized to the temperature measured most immediately prior to injection (Krajewski-Hall et al., 2018; Krull et al., 2017). Images captured on the last day of the experiment were quantified for T_Skin_. Previously, Krajewski-Hall et al. (2018) utilized a threshold of 30 °C, above which mice were removed from analyses of senktide-induced T_Skin_ measurements. Based upon our own preliminary studies, we determined that T_Skin_ exceeding 27 °C prior to peripheral injection is indicative of measurement-induced stress and/or increased activity (data not shown), thereby obfuscating effects of senktide on tail vasodilation as measured by thermal imaging. Therefore, based on this previous literature and our own preliminary studies, mice were removed from vehicle (*n* = 1, DPN) or senktide (n = 1, DPN) analyses if their T_Skin,_ Baseline was above 27 °C (Krajewski-Hall et al., 2018). Additionally, mice were removed from senktide challenge analyses if they did not exhibit tail rattles or cold-seeking behaviors, such as pushing bedding aside to expose the cool bottom of the cage (n = 1, Vehicle), both of which are stereotypical responses to senktide treatment (Krajewski-Hall et al., 2018; Krull et al., 2017).

### 2.6. Open field test

The open field (OF) test assessed locomotor and anxiety-like behaviors (Li et al., 2014; Lund et al., 2005; Oyola et al., 2012; Rocha et al., 2005). The OF arena consisted of an empty white testing box (60 cm × 60 cm × 47 cm) that was divided into a 5 × 5 grid of squares (12 cm × 12 cm). This grid included an outer zone (16 squares total), a middle zone (8 squares total), and a center zone (1 square total) (Fig. 2). Mice were transported to a holding room immediately following gavage and remained in the room for 1 h prior to testing to acclimate to a new environment. Mice were placed in the lower center of the OF arena, facing the bottom wall, and given 10 min to explore. ANY-maze software (Stoelting) automatically tracked the center of each mouse’s body and quantified distance traveled in, time spent in, and number of entries into each zone. The number of fecal boli in each zone, as well as the number of bouts and time spent both grooming/barbering and rearing, were manually quantified. After testing, each mouse was immediately returned to its home cage and removed from the testing room. The OF arena was cleaned with 70% ethanol between each test.

### 2.7. Elevated plus maze

The elevated plus maze (EPM) was also used to measure anxiety-like behavior (Frick et al., 2000; Lund et al., 2005; Oyola et al., 2012). The open arms of the EPM apparatus (30 cm × 5 cm) consisted of an opaque white Plexiglas floor with a clear lip (0.5 cm) attached to the sides of each arm to prevent mice from falling off the apparatus (Fig. 2). The closed arms (30 cm × 5 cm × 15 cm) consisted of opaque black Plexiglas walls and a gray floor. Mice were transported to a holding room, gavaged, and given 1 h to acclimate to the new environment before moving to the testing room and being placed in the EPM. Mice were placed in the center of the maze, facing the upper open arm, and behavior was recorded via ANY-maze for 10 min. Following completion of EPM testing, the number of fecal boli, number of entries into, and time spent in each set of arms and the center zone were quantified. An entry into either set of arms was determined as a mouse having all four paws within an arm. Otherwise, mice were considered to be in the center zone. Time spent peeking into open arms was also quantified, defined as any time the mouse either oriented its head and up to three paws into an open arm or was looking over the side of the apparatus. Mice were immediately placed back into their home cages and removed from the room after testing, and the EPM was cleaned with soap and water between each test. One mouse (EGX358) was removed from analyses because it fell off of the apparatus during the test.

### 2.8. Tail suspension test

The tail suspension test (TST) assessed depression-like behavior (Bernardi et al., 1989; Can et al., 2011). The TST apparatus was made of opaque white Plexiglas and consisted of three chambers (10 in × 10 in × 18 in) (Fig. 2). A tube was threaded through a hole in the ceiling of each chamber. C57BL/6 mice are known to climb their tails when suspended in this manner, making it difficult to quantify mobility and immobility behaviors (Can et al., 2011). Therefore, a small piece of tubing was placed around the base of each mouse’s tail to effectively eliminate tail climbing (Can et al., 2011). Each mouse was suspended in the chamber by its tail, which was taped to the tube 1 cm from the tail tip and with the mouse’s ventral side facing the camera. The mouse’s behavior was recorded via ANY-maze for 6 min. Following testing, tubing was removed from each mouse’s tail, and mice were returned to their home cages and removed from the room. The TST apparatus was cleaned with soap and water between each test. After testing, the number of fecal boli, time spent immobile, and latency to first bout of immobility were measured. Immobility was defined when the mouse remained motionless or made only those movements needed to reorient its body, such as small front paw movements, slow adjustments, or sniffing. Mobility was defined as additional movements, such as full-body movement, rear paw movement, forceful front paw movement, and the mouse climbing the fur on its abdomen. To ensure accurate scoring, all tests were scored by a single researcher blind to treatment, and each test was scored until two independent immobility scores were within 3 s of each other. The average of these two scores was used as the immobility score for each mouse.

### 2.9. Forced swim test

The forced swim test (FST) was used to measure depression-like behavior (Bekku and Yoshimura, 2005; Can et al., 2011; Estrada-Camarena et al., 2003; Li et al., 2014; Okada et al., 1997; Rocha et al., 2005). The FST apparatus consisted of a glass cylinder (14 in × 6 in diameter) filled with water (20 cm depth) to prevent mice from supporting themselves by touching the cylinder bottom with their paws (Fig. 2). Water temperature was measured between each test and maintained at 23 ± 2 °C, and water was changed after three mice completed testing. During the FST, each mouse was gently placed into the water, such that its head was not submerged, and swimming behavior was recorded for 6 min in ANY-maze. After testing, the mouse was removed from the cylinder, dried with paper towels, and returned to its home cage. The home cage was then removed from the testing room and placed on a heating pad for 20 min. The number of fecal boli was recorded and cleaned from the glass cylinder after each mouse completed the FST. The first 2 min of each recording were used to acclimate each mouse to the water. The last 4 min were scored for time spent immobile and latency to first bout of immobility by a single researcher who was blind to treatment groups. Immobility was defined as the lack of movement (floating) or only those movements necessary to rectify the mouse’s upright position. Mobility was defined as additional movements, such as swimming and attempting to climb the cylinder wall. As with the TST, each test was scored until two independent immobility scores were within 3 s of each other. The average of the two scores was used as the immobility score for each mouse.

### 2.10. Object placement and object recognition

The object placement (OP) and object recognition (OR) tests (Fig. 2) assessed spatial and object recognition memory, respectively (Boulware et al., 2013; Fernandez et al., 2008; Fortress et al., 2013a; Fortress et al., 2013b; Hanson et al., 2018; Kim et al., 2016; Tuscher et al., 2016b). Briefly, one week after the FST, a Lego Duplo block was placed in the home cage of each mouse for three days to expose it to an object. Then, each mouse was habituated to an empty OF box (60 cm × 60 cm × 47 cm) for 5 min/day for two days. The day after the second habituation, the mouse was placed in the OF box for 2 min before training with objects. After the 2 min accustomization phase, the mouse was returned to its home cage while two identical objects were positioned 5 cm from the upper left and right corners of the OF arena. The mouse was then returned to the OF box and given 20 min to accumulate 30 s of exploration time with the objects, defined as any time when the mouse was adjacent to a given object with its nose and/or front paws directed at and/or touching the object. Exploration was scored live in ANY-maze by a researcher blind to treatment. Mice underwent OP training and testing prior to OR training and testing.

OP and OR were tested 24 h and 48 h post-training, respectively. At these time points, OVX mice treated acutely with E_2_, DPN, or EGX358, but not vehicle, immediately post-training spend significantly more time than chance (15 s) with the moved or novel objects, respectively (Boulware et al., 2013; Hanson et al., 2018; Tuscher et al., 2019; Kim et al., 2016). During both OP and OR testing, as in training, mice were given 20 min to accumulate 30 s of exploration between the objects, as scored live in ANY-maze. Mice that remember the location and identity of the training objects should spend significantly more time than chance with the moved and novel objects in OP and OR, respectively. Following testing, each mouse was removed from the OF arena and returned to its home cage. The OF arena was cleaned with 70% ethanol between mice after each portion of both OP and OR. Mice were removed from OP (*n* = 1, DPN; *n* = 2, EGX358) or OR (n = 1, Vehicle; n = 1, DPN) analyses if they did not reach >27 s of exploration time with objects during training and 30 s during testing.

### 2.11. Statistical analyses

Prior to statistical analyses of treatment effects, outliers, assessed as any data points > 2 standard deviations from the group mean, were removed from each data set. Task-specific exclusions are described above. Finally, one E_2_-treated and two DPN-treated mice were removed prior to the conclusion of the study and euthanized due to declining health, as indicated by aberrant amounts of barbering or >5% weight loss between weeks. Therefore, sample sizes are not equal across all test parameters in each measurement, although sample sizes are noted in-text.

All statistical tests were conducted using GraphPad Prism 8 (La Jolla, CA). All anxiety-and depression-like measurements, and final T_Skin_ measurements were analyzed by one-way analysis of variance (ANOVA), followed by Tukey post hoc tests on main effects of group. Body weight measurements and ΔT_Skin_ measurements during vehicle and senktide challenge were analyzed by mixed-effects two-way ANOVA (treatment x time since OVX, and treatment x time since SC injection, respectively) due to removal of outliers at individual time points, followed by Tukey post hoc tests. For memory in the OP and OR tests, two analyses were performed. To assess memory within each group, one-sample *t-*tests were used to compare time spent with the moved or novel object, respectively, to chance (15 s). One-way ANOVAs were conducted to compare treatment groups to one another, followed by Tukey’s post hoc tests on main effects of treatment. Significance was defined at *p* < 0.05. Effect sizes were calculated as Cohen’s d for significant *t-*tests and eta squared (η^2^) for significant ANOVAs.

## 3. Results

### 3.1. Long-term oral administration of a highly selective ERβ agonist, EGX358, did not adversely affect overall health or body weight

We first sought to ensure that long-term administration of EGX358 does not cause adverse health effects, as measured by disheveled physical appearance or premature death. Young female mice were OVXed (*n* = 40). Following recovery from surgery (Fig. 1), mice were gavaged with vehicle (*n* = 10), E_2_ (n = 10), DPN (n = 10), or EGX358 (n = 10) for 9 weeks. Throughout the duration of this study, three mice were removed due to excessive barbering, weight loss, or poor apparent health. Two DPN-treated mice were removed and euthanized due to weight loss and poor physical appearance, and one E_2_-treated mouse was removed and euthanized due to excessive barbering, but no vehicle-or EGX358-treated mice were removed from this study. The lack of poor outcomes in the EGX358 group suggests that long-term oral administration of EGX358 has minimal negative impact on overall health, as observed by physical appearance and measured by weight status.

**Fig. 3.**
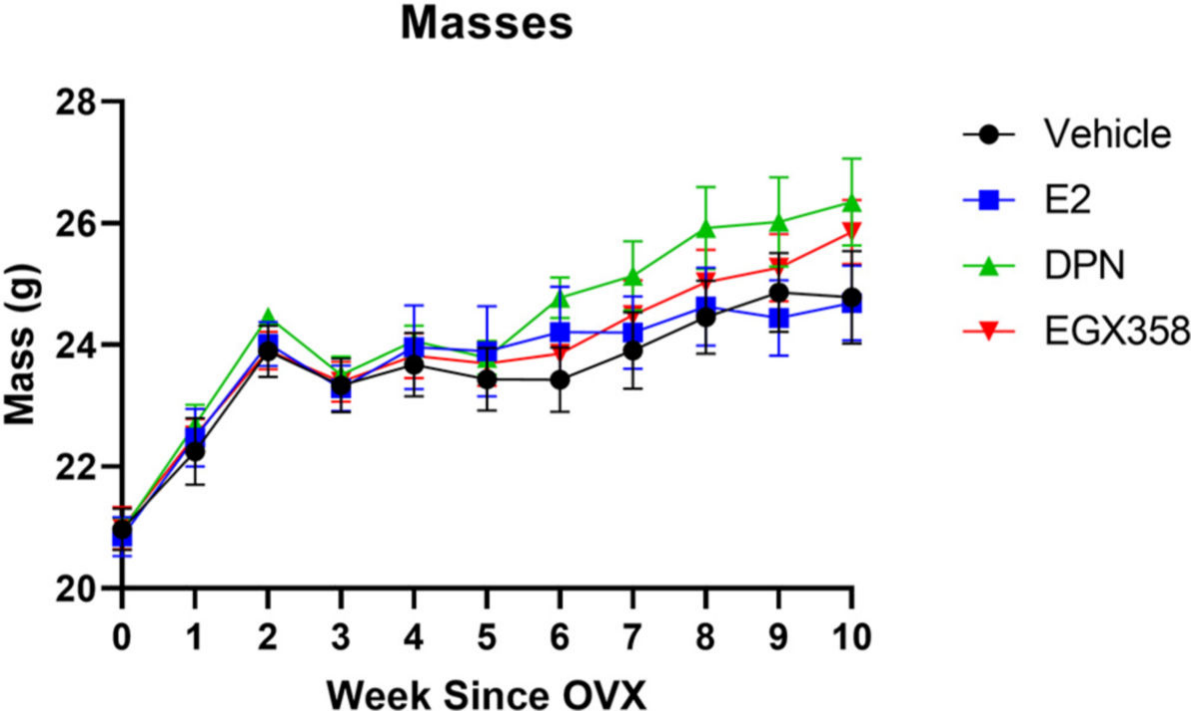
Long-term treatment with EGX358 did not alter weight gain following ovariectomy. Young female C57BL/6 mice were bilaterally ovariectomized and given 1 week to recover. Following recovery, mice began daily oral gavage treatment with vehicle (10% DMSO; *n* = 10), E_2_ (n = 10), DPN (n = 10), or EGX358 (n = 10). Daily treatment continued throughout the duration of the experiment, for 64 days in total. Mice were weighed immediately prior to ovariectomy surgery and at the beginning of every week thereafter to both adjust treatment volume, as well as examine the effects of chronic EGX358 treatment on body weight following ovariectomy. There was a significant increase in body weight over the course of the experiment (*p* < 0.0001) that did not significantly differ among the groups at any time point. DMSO, dimethyl sulfoxide; E_2_, 17β-estradiol; DPN, diarylpropionitrile. Error bars represent mean +/−standard error of the mean (SEM).

Mice were also weighed weekly to adjust treatment volume and measure effects of long-term treatment with EGX358 on weight gain following OVX, a commonly documented phenomenon post-OVX that is reduced by E_2_ when compared to vehicle treatment (Couse and Korach, 1999; Sibonga et al., 1998). Mixed-effects two-way ANOVA (treatment x time since OVX) analysis revealed a significant effect of time (*F*_(4.102, 138.7)_ = 103.6, *p* < 0.0001, η^2^ = 0.8815), but not of treatment (*F*_(3, 36)_ = 0.5673, *p* = 0.6401). Although the time x treatment interaction was significant (*F*_(30, 338)_ = 1.644, *p* = 0.0202, η^2^ = 0.1023), Tukey’s post hoc tests indicated no significant between-group differences at any time point (Fig. 3). This interaction appears to be driven by the DPN-treated group, which weighed somewhat more on average than the other groups beginning 6 weeks post-OVX, although the lack of significant post hoc analyses shows this to be a rather weak trend. Collectively, these results suggest that long-term oral EGX358 administration did not adversely affect overall health, nor did it affect weight gain relative to vehicle treatment.

### 3.2. EGX358, DPN, and E_2_ similarly alleviated an acute drug-induced increase in tail skin temperature

Although our group has shown that acute post-training administration of EGX358 promotes memory consolidation in OVX mice (Hanson et al., 2018), we have yet to study the effects of EGX358 on other preclinical indices of menopausal symptoms. Here, we aimed to determine whether long-term oral treatment with EGX358 at the lowest memory-enhancing dose observed in our previous study could reduce hot flash-, anxiety-, and depression-like symptoms and enhance memory formation. Previous studies have demonstrated that long-term treatment with E_2_ or ERβ-selective phytoestrogen diets can prevent increases in T_Skin_ in rats and mice following OVX (Opas et al., 2004; Zhao et al., 2011), and that this effect is seen in ERα knockout mice (Opas et al., 2006). However, this model does not recapitulate the sudden and transient nature of hot flashes, which are driven by disrupted neurokinin B signaling in the median preoptic area due to the loss of circulating estrogens (Padilla et al., 2018; Rance et al., 2013; Tan et al., 2016). A more recently developed method for vasomotor symptom induction is the administration of a tachykinin 3 receptor agonist, senktide, which elicits a transient increase in T_Skin_ that is prevented by long-term E_2_ administration (Krajewski-Hall et al., 2018; Krull et al., 2017). Here, we used this method to model hot flash-like symptoms and their treatments.

**Fig. 4.**
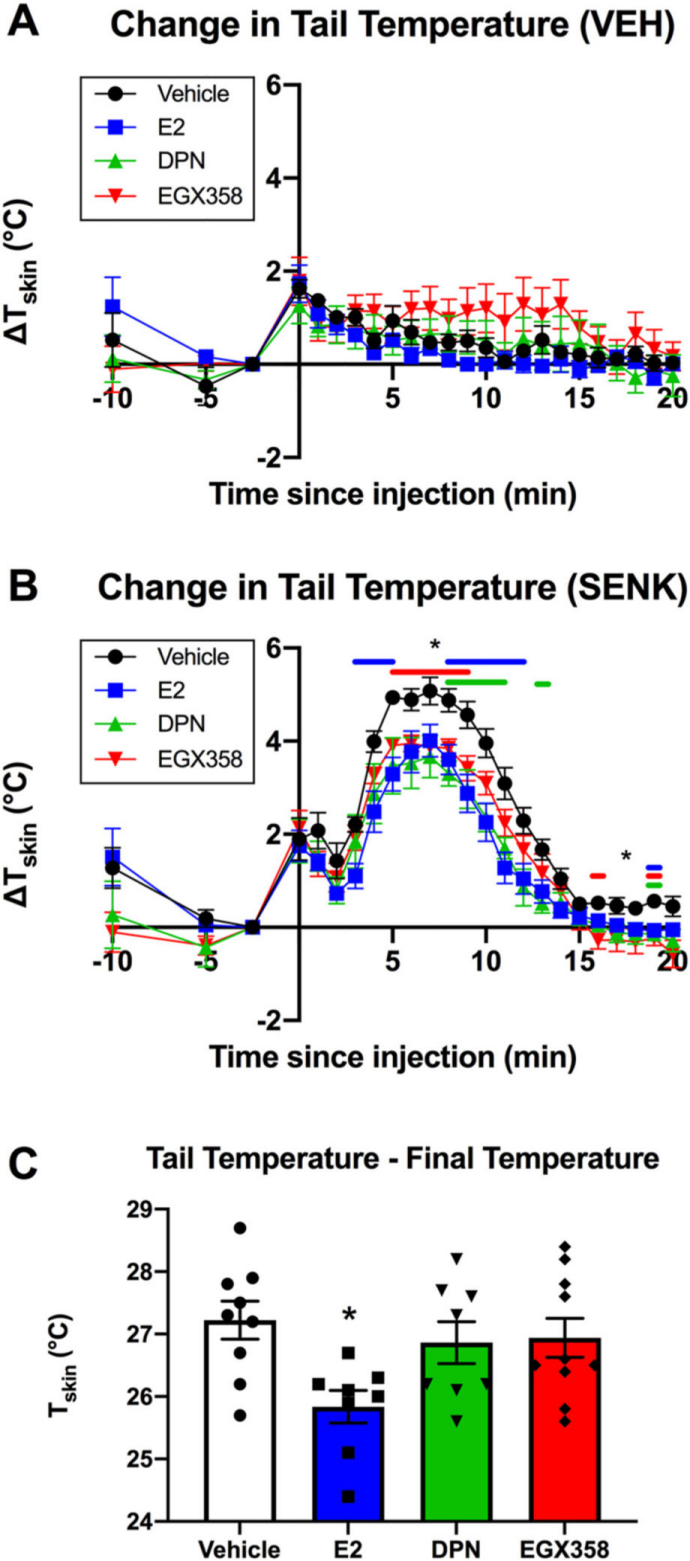
Long-term treatment with EGX358 reduced senktide-mediated increases in tail skin temperature. Following 2 weeks of daily gavage with vehicle (n = 10), E_2_ (n = 10), DPN (*n* = 8), or EGX358 (n = 10), mice were evaluated in a senktide-mediated model of vasodilation. Mice were tested twice, once after being injected subcutaneously with 0.9% saline and once after senktide injection, to ensure that senktide-mediated ΔT_Skin_ could not be attributed to the injection process. A) Baseline T_Skin_ was thermally imaged for 10 min, after which mice were injected subcutaneously with 0.9% saline and then were imaged for another 20 min. Compared to the baseline T_Skin_ measured 2.5 min prior to injection, subcutaneous injection caused a modest increase in T_Skin_ that returned to baseline within 20 min in all treatment groups (*p* < 0.0001). An interaction between treatment and time since injection (*p* = 0.0227) was observed, such that EGX358-treated mice tended be modestly warmer than other mice at 5–15 min post-injection, although post hoc analyses revealed no significant differences between treatment groups at any time point. B) In all treatment groups, subcutaneous injection of senktide caused a transient, significant increase in T_Skin_ compared to baseline (*p* < 0.0001). However, an effect of treatment (*p* = 0.0041) and an interaction between treatment and time since injection (*p* < 0.0001) were observed. E_2_, DPN, and EGX358 reduced ΔT_Skin_ due to injection compared to vehicle, such that E_2_-treated mice had significantly lower ΔT_Skin_ compared to vehicle-treated mice 3–5, 8–12, and 19 min post-injection (blue lines, *p < 0.05); DPN-treated mice had significantly lower ΔT_Skin_ compared to vehicle 8–11, 13, and 19 min post-injection (green lines, *p < 0.05); and EGX358-treated mice had significantly lower ΔT_Skin_ due to injection compared to vehicle 5–9, 16, and 19 min post-injection (red lines, *p < 0.05). C) T_Skin_ was measured on the final day of the experiment to determine the effects of long-term treatment on baseline T_Skin_. E_2_-treated mice had significantly lower T_Skin_ compared to vehicle-treated mice after 63 days of treatment (*p* = 0.0221). E_2_, 17β-estradiol; DPN, diarylpropionitrile; T_Skin_, tail skin temperature; ΔT_Skin_, change in tail skin temperature relative to baseline. Error bars represent mean ± SEM. *p < 0.05, compared to vehicle.

Sample sizes at the start of the study were *n* = 10/group. As described above and where appropriate in the following sections, some mice were excluded from data analyses due to various issues. As such, sample sizes below reflect the number of mice in each group included in the data analyses. Eleven days after ovariectomy, mice began oral gavage treatment. After two weeks of treatment, mice gavaged with vehicle, E_2_, DPN or EGX358 were then injected SC with vehicle solution 1 h later to determine possible effects of injection procedures on T_Skin_ (Figs. 1, 2). Sample sizes were as follows: vehicle (*n* = 10), E_2_ (n = 10), DPN (*n* = 7), and EGX358 (n = 10). As others have demonstrated that ambient room temperature affects T_Skin_ in OVX mice (Krajewski-Hall et al., 2018), the testing room temperature was measured at the beginning of each mouse’s vehicle challenge test session. During testing, room temperature ranged between 22.0 and 22.5 °C. In the vehicle challenge, a modest and transient increase in T_Skin_ relative to baseline (ΔT_Skin_) was observed immediately after SC injection that did not significantly differ between groups (Fig. 4A). Mixed-effects two-way ANOVA (treatment x time since SC injection) revealed a main effect of time (*F*_(4.731, 154.3)_ = 9.169, *p* < 0.001, η^2^ = 0.2959), but no main effect of treatment (*F*_(3, 34)_ = 1.070, *p* = 0.3746). Although the time x treatment interaction was significant (*F*_(69, 750)_ = 1.393, *p* = 0.0227, η^2^ = 0.6557), Tukey’s post hoc tests indicated no significant between-group differences at any time point, despite modestly higher ΔT_Skin_ in EGX358-treated mice between 5 and 15 min following SC injection. These results suggest that the injection process itself caused a modest, but transient, increase in T_Skin_ that was not significantly influenced by E_2_ or ERβ agonist treatment.

Four days later, the mice were gavaged and injected SC with senktide 1 h later (Figs. 1, 2). Room temperature measured before each mouse’s senktide challenge ranged between 21.4 and 22.1 °C. Mixed-effects two-way ANOVA (treatment x time since injection) revealed main effects of time (*F*_(6.052, 188.4)_ = 147.7, *p* < 0.0001, η^2^ = 0.8560) and treatment (*F*_(3,32)_ = 5.381, *p* = 0.0041, η^2^ = 0.0155), and a time x treatment interaction (*F*_(69, 716)_ = 1.895, *p* < 0.0001, η^2^ = 0.1252; Fig. 4B). Post hoc tests revealed that E_2_-treated mice had significantly reduced ΔT_Skin_ (change in TS_kin_ relative to pre-injection baseline T_Skin_) compared to vehicle-treated mice 3–5, 8–12, and 19 min post-injection (*p* < 0.05). DPN significantly reduced ΔT_Skin_ compared to vehicle 8–11, 13, and 19 min post-injection (*p* < 0.05). Finally, EGX358 significantly reduced ΔT_Skin_ compared to vehicle 5–9, 16, and 19 min post-injection (*p* < 0.05). No significant differences among the E_2_, DPN, or EGX358 groups were revealed at any time point in this test. These results demonstrate similarly beneficial efficacy of E_2,_ DPN, and EGX358 in reducing vasodilation induced by activation of neurokinin B receptors.

Finally, we examined whether long-term treatment could prevent the gradual increase in T_Skin_ associated with long-term OVX (Opas et al., 2004; Rance et al., 2013; Zhao et al., 2011). The day following completion of OR testing (day 63 of treatment), mice were gavaged and thermally imaged in their home cages 1 h later (Figs. 1, 2). Sample sizes were as follows: vehicle (*n* = 9), E_2_ (*n* = 8), DPN (n = 8), and EGX358 (*n* = 10). Throughout treatment and imaging, the temperature of the colony room in which observations were made ranged between 23.3 and 23.8 °C. One-way ANOVA revealed a significant effect of treatment on baseline TSkin (*F*_(3,31)_ = 3.691, *p* = 0.0221, η^2^ = 0.2632; Fig. 4C), with post hoc analyses showing that only E_2_-treated mice had significantly lower baseline T_Skin_ compared to vehicle-treated mice (*p* < 0.05).

Collectively, our results indicate that a relatively low dose of EGX358 reduced transient increases in tail skin vasodilation induced by neurokinin B signaling to similar extents as E_2_ and DPN. However, only E_2_, but not ERβ agonists alone, reduced baseline T_Skin_ when compared to vehicle treatment.

### 3.3. EGX358 and other estrogenic compounds did not affect anxiety-like or locomotor behaviors

Long-term OVX in rats and mice is associated with increased anxiety-like behaviors when compared to sham-operated controls, and these behaviors are reduced by systemic administration of estrogenic compounds, including E_2_ and DPN, relative to vehicle treatment (Frye and Walf, 2004; Lagunas et al., 2010; Li et al., 2014; Lund et al., 2005; Oyola et al., 2012; Walf and Frye, 2005a, 2006). Therefore, we next sought to determine the effects of EGX358 on anxiety-like behaviors. The day after completion of the senktide tail vasodilation test (day 23 of treatment), mice were gavaged with vehicle (*n* = 10), E_2_ (n = 10), DPN (*n* = 8), or EGX358 (n = 10) (Figs. 1, 2). One hour later, mice were placed into an empty OF box and the time, distance traveled, fecal boli, and entries in the outer, middle, and center zones were recorded. One-way ANOVAs revealed no significant treatment effects for fecal boli in the outer, middle, and center zones or for total fecal boli (Table 1). Similarly, total distance traveled, distance in the outer, middle, and center zones, and entries into the outer, middle, and center zones did not differ significantly among the groups (Table 1). Although the groups did not differ in time spent in the outer or middle zones, the treatment effect was significant for time spent in the center zone (*F*_(3, 33)_ = 4.269, *p* = 0.0118, η^2^ = 0.2796; Table 1). Post hoc analyses revealed that E_2_-treated mice spent significantly more time in the center zone than all other treatment groups (all *p* values <0.05).

We also measured time and bouts spent grooming/barbering and rearing. The main effect of treatment was significant for time spent grooming/barbering (*F*_(3, 31)_ = 3.506, *p* = 0.0268, η^2^ = 0.2534; Table 1). Although post hoc tests did not indicate significant differences between any of the treatment groups, the treatment effect may have been driven by modestly reduced time grooming/barbering in E_2_-and EGX358-treated mice relative to vehicle-and DPN-treated mice. The groups also differed significantly in time spent rearing (*F*_(3, 33)_ = 4.278, *p* = 0.0117, η^2^ = 0.2800; Table 1), due to increased rearing in E_2_-treated mice relative to vehicle. No significant effects of treatment were observed for bouts of grooming/barbering or rearing (Table 1).

Next, we measured the effects of long-term treatment on anxiety-like behaviors in the EPM. One day after completion of the OF test (day 25 of treatment), mice were gavaged with vehicle (*n* = 10), E_2_ (n = 10), DPN (*n* = 8), or EGX358 (*n* = 9) and were placed into the EPM apparatus 1 h later (Figs. 1, 2). For data analysis, the EPM was divided into three zones: open arms, closed arms, and center. The time spent, number of entries, and number of fecal boli in each zone was recorded, as were total entries into all zones and total boli. However, no treatment differences were observed in any of these measures (Table 1). We also assessed “peeking” behavior by quantifying the number of peeking bouts and time spent peeking into the open arms or over the edge of the apparatus. As with the other EPM measures, no significant treatment differences were observed in either of these measurements (Table 1).

Together, our results suggest a modest anxiolytic effect of long-term oral E_2_ treatment, but not ERβ agonism, at least at the doses tested, in young ovariectomized mice. Additionally, our results suggest no effect of E_2_ treatment or ERβ agonism on locomotor behaviors.

### 3.4. EGX358 and other estrogenic compounds did not affect depression-like behaviors

As with anxiety-like behaviors, previous research indicates that OVX mice and rats display greater depression-like behaviors when compared to sham-operated controls, and that these behaviors are reduced by administration of estrogenic compounds, including E_2_ and DPN, compared to vehicle treatment (Li et al., 2014; Rocha et al., 2005; Walf et al., 2004; Walf and Frye, 2006, 2007). Therefore, we next examined potential effects of long-term EGX358 treatment on depression-like behaviors in the TST and FST. One day following completion of EPM testing (day 27 of treatment), mice were gavaged with vehicle (*n* = 10), E_2_ (n = 10), DPN (n = 8), or EGX358 (n = 10) and were suspended by their tails 1 h later (Figs. 1, 2). The time spent immobile, latency to first bout of immobility, and number of fecal boli produced were recorded. One-way ANOVAs revealed no significant treatment effects on any measure (Table 2). The next day (day 30 of treatment), mice were gavaged and placed into the FST cylinder 1 h later. As in the TST, time spent immobile, latency to first bout of immobility, and number of fecal boli were recorded. Similar to the TST, no treatment effects were significant for any measure (Table 2). Collectively, these findings suggest that long-term EGX358 treatment at the current dose does not affect depression-like behaviors in a mouse model of menopause.

These findings suggest no effect of daily oral E_2_ treatment or ERβ agonism, at least at the doses tested, on depression-like behaviors in young ovariectomized mice.

### 3.5. EGX358 enhanced both spatial and object recognition memory

Our laboratory has previously demonstrated that a single post-training intraperitoneal (i.p.) injection of E_2_ enhances spatial memory consolidation in the Morris water maze and recognition memory consolidation in OR (Gresack and Frick, 2006a, 2006b), and that a single post-training administration (i.p. injection or oral gavage) of DPN or EGX358 enhances memory consolidation in both OR and OP in young ovariectomized mice (Hanson et al., 2018). Here, we aimed to determine whether long-term daily gavage of these compounds at the same doses would enhance memory in the OP and OR tasks. OP training and testing began on day 41 of treatment and OR training and testing started on day 52 of treatment (Figs. 1, 2).

**Fig. 5.**
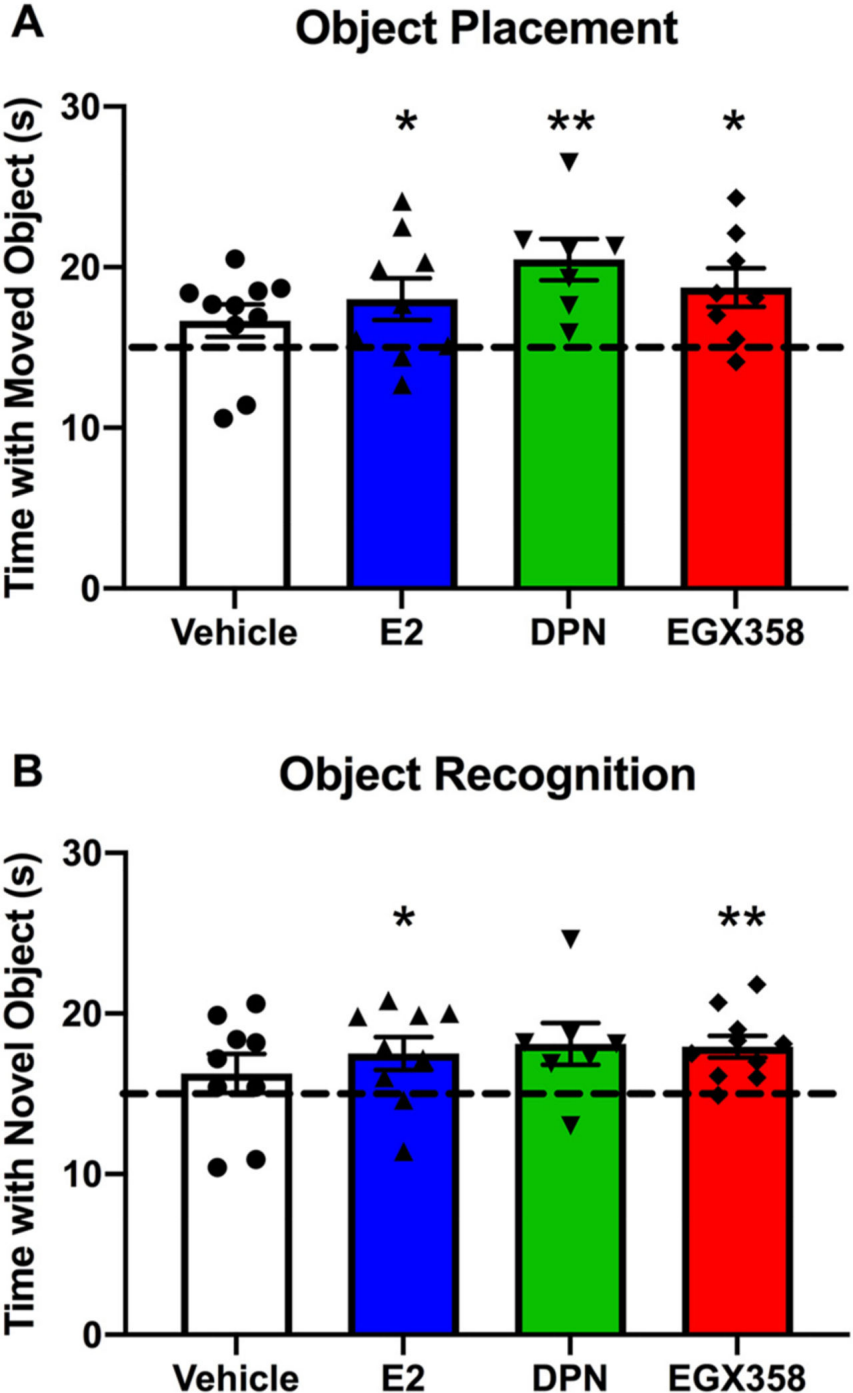
Long-term treatment with EGX358 enhanced spatial and object recognition memory. Following completion of forced swim testing, mice continued daily gavage of vehicle (n = 10), E_2_ (*n* = 9), DPN (*n* = 7), or EGX358 (n = 10) without being tested for 7–8 days, after which they began training and testing in OP and, subsequently, OR. A) Mice were gavaged 1 h prior to training and testing phases of the OP task. Mice treated with E_2_, DPN, or EGX358, but not vehicle, spent significantly more time than chance (dashed line at 15 s) with the moved object during the testing phase of OP. B) Similar to OP, mice gavaged with E_2_ or EGX358 1 h prior to training, 24 h later, and 1 h prior to testing spent significantly more time than chance with the novel object during the testing phase of OR. E_2_, 17β-estradiol; DPN, diarylpropionitrile; OP, object placement; OR, object recognition. Error bars represent mean ± SEM. *p < 0.05, compared to chance.

One hour prior to OP training, mice were gavaged with vehicle (n = 10), E_2_ (*n* = 9), DPN (*n* = 7), or EGX358 (n = 8) (Fig. 2). Twenty-three hours later, mice were again gavaged with their respective treatments and tested 1 h later. Learning within a group was demonstrated with one-sample *t*-tests, which revealed that mice receiving E_2_ (*t*_(8)_ = 2.320, *p* = 0.0489, *d* = 0.7728; Fig. 5A), DPN (*t*_(6)_ = 4.266, *p* = 0.0053, *d* = 1.6138), or EGX358 (*t*_(7)_ = 3.117, *p* = 0.0169, *d* = 1.1026) spent significantly more time than chance (15 s) with the moved object, whereas vehicle-treated mice did not (*t*_(9)_ = 1.654, *p* = 0.1326). Although a one-way ANOVA revealed no main effect of treatment (*F*_(3, 30)_ = 1.710, *p* = 0.186), the pattern of within-group learning suggests that long-term treatment with E_2_, DPN, or EGX358 enhanced spatial memory formation in the OP task.

OR was conducted similarly, except that the delay between training and testing was 48 h instead of 24 h, requiring mice to be gavaged with vehicle (n = 9), E_2_ (n = 9), DPN (n = 7), or EGX358 (n = 10) three times: 1 h prior to training, and again 24 and 48 h following the first treatment (Fig. 2). Mice were tested 1 h after the third gavage. One-sample *t*-tests revealed that whereas E_2_-(*t*_(8)_ = 2.439, *p* = 0.0406, *d* = 0.8128) and EGX358-treated (*t*_(9)_ = 4.342, *p* = 0.0019, *d* = 1.3732; Fig. 5B) mice spent more time than chance with the novel object, vehicle-(*t*_(8)_ = 1.045, *p* = 0.3267) and DPN-treated (*t*_(6)_ = 2.400, *p* = 0.0533) mice did not. Although the DPN group mean was similar to that for EGX358, its variability was higher, likely due to its smaller sample size. Again, the main effect of treatment was not significant (*F*_(3, 31)_ = 0.6365, *p* = 0.5972), but the within-subjects analyses suggest that long-term E_2_ or EGX358 treatment enhanced object recognition memory.

Together, these findings suggest that long-term oral gavage of E_2_ and EGX358 enhances spatial and object recognition memory in young ovariectomized mice, with DPN providing some similar benefits.

## 4. Discussion

We and others have previously demonstrated roles for ERβ signaling in enhancing memory processes (Boulware et al., 2013; Hanson et al., 2018; Jacome et al., 2010; Kim and Frick, 2017; Pereira et al., 2014; Walf et al., 2006), reducing anxiety-and depression-like behaviors (Li et al., 2014; Lund et al., 2005; Oyola et al., 2012; Rocha et al., 2005; Walf et al., 2004, 2008; Walf and Frye, 2005a, 2005b), and reducing vasomotor symptoms (Opas et al., 2006; Zhao et al., 2011) in OVX rodents. However, the present study is the first to examine the effects of long-term oral ERβ agonists on preclinical indices of menopausal symptoms in OVX mice. This work is particularly notable for its evaluation of EGX358, which is 750-fold more selective for ERβ over ERα (Hanson et al., 2018), and therefore, the most selective ERβ synthetic agonist available to date. Here, we showed that daily gavage of EGX358, DPN, and E_2_ reduced senktide-induced increases in T_Skin_ after ~2.5 weeks of treatment, and that 9 weeks of E_2_, but not ERβ agonist alone, treatment reduced baseline T_Skin_ compared to vehicle treatment. Next, we demonstrated that, although EGX358, DPN, and E_2_ had largely no effects on anxiety-or depression-like behaviors, daily gavage for 8–9 weeks enhanced spatial and object recognition memory in the OP and OR tasks. Importantly, long-term oral administration of EGX358 did not result in premature death or adverse health effects in any mice, although estrogenic treatment failed to prevent weight gain compared to vehicle. Collectively, these results provide promising evidence that long-term administration of selective ERβ agonists like EGX358 may be effective treatment options for some menopausal symptoms.

### 4.1. Effects on vasodilation

Our data showing that both DPN and our novel, highly selective ERβ agonist, EGX358, prevent senktide-mediated increases in T_Skin_ are the first to demonstrate a role for ERβ activation in this model. Previous findings have demonstrated that systemic senktide administration produces rapid, transient increases in T_Skin_ in male and gonadally-intact and OVX female mice (Krajewski-Hall et al., 2018; Krull et al., 2017). These effects are mediated by the binding of senktide to tachykinin 3 receptors in the median preoptic area of the hypothalamus (Mittelman-Smith et al., 2015; Padilla et al., 2018), thereby activating warm-sensitive neurons in this region and initiating vasodilation and cold-seeking behaviors in rodents (Krull et al., 2017; Rance et al., 2013; Tan et al., 2016). Importantly, previous studies have also demonstrated that tachykinin 3 receptor-mediated tail vasodilation responses are reduced in intact compared to OVX female mice (Padilla et al., 2018) and following senktide administration in OVX mice treated chronically with E_2_ via silastic capsule compared to vehicle-treated mice (Krajewski-Hall et al., 2018). We add to this growing body of literature by demonstrating that long-term treatment with ERβ agonists can mitigate senktide-induced increases in T_Skin_. Importantly, E_2_ (0.2 mg/kg), DPN (0.05 mg/kg), and EGX358 (0.5 mg/kg) all mitigated senktide-mediated changes in T_Skin_ compared to vehicle treatment, suggesting a protective role of ERβ signaling against neurokinin B-mediated hot flash symptoms. Although T_Skin_ increased modestly and transiently after vehicle injection, it is highly unlikely that this increase influenced the effects of estrogenic compounds on T_Skin_. Importantly, the ΔT_Skin_ increase following senktide injection was more than 2-fold higher than after vehicle injection, with ΔT_Skin_ of under 2 °C after vehicle injection and of 4–5 °C after senktide injection. Additionally, only senktide-treated mice exhibited characteristic tail rattles and expressed cold-seeking behaviors, such as moving cage bedding around to create bare, cool spots, where they would reside for much of the test, behaviors that were unaffected by estrogenic compound treatment. Others have made similar observations following senktide treatment in mice (Krajewski-Hall et al., 2018; Krull et al., 2017). These quantitative and qualitative differences suggest that the estrogenic compounds specifically mitigated the vasodilatory response to senktide, not a more general reaction to the injection procedure. As such, our data indicate that long-term oral treatment with E_2_ or ERβ agonists such as EGX358 can reduce vasodilation in a neurokinin B-mediated model of hot flashes.

Interestingly, nine weeks of ERβ agonist treatment did not affect baseline T_Skin_. Long-term OVX is a common method for modeling vasomotor symptoms and their treatment in rodents, as this surgical method induces gradual increases in tail vasodilation (Berendsen et al., 2001; Dacks and Rance, 2010; Kobayashi et al., 1995; Opas et al., 2004; Zhao et al., 2011), and long-term treatment with estrogenic compounds often prevents this elevation in temperature in both mice and rats (Berendsen et al., 2001; Bowe et al., 2006; Kobayashi et al., 1995; Opas et al., 2004, 2006; Zhao et al., 2011). We recapitulate these findings by demonstrating that E_2_ (0.2 mg/kg) administered for 9 weeks via gavage reduces baseline T_Skin_ in OVX mice relative to vehicle-treated mice. However, DPN (0.05 mg/kg) and EGX358 (0.5 mg/kg) treatment did not alter T_Skin_ relative to vehicle treatment at this time point. These findings contrast with previous studies demonstrating that ERβ-selective phytoestrogen diets and DPN injections can reduce increases in T_Skin_ compared to vehicle treatment following OVX in both rats and mice (Bowe et al., 2006; Opas et al., 2006; Zhao et al., 2011). However, the long-term design of our study, in addition to the relatively low dosage of both DPN and EGX358, may have contributed to these contrasting results. For example, Bowe et al. (2006) showed that four days of subcutaneous injections of DPN at 0.6 mg/kg, a dose twelve-fold greater than that administered here, lowered ΔT_Skin_ relative to first-day measurements compared to vehicle-treated OVX rats. As our chosen EGX358 dose is proportional to our DPN dose, based upon the previously established relative potency of the compounds (Hanson et al., 2018), a higher dose of EGX358 may have more effectively lowered ΔT_Skin_ relative to vehicle treatment. Additionally, previous studies have demonstrated that T_Skin_ is a circadian rhythm, and that sensitivity to treatment with estrogenic compounds may be higher during the dark-phase than the light-phase (Girbig et al., 2012; Williams et al., 2010). As our measurements were made during the light-phase and only at the end of the study, it is possible that effects of daily EGX358 treatment on baseline T_Skin_ may have been missed or obscured. Follow-up studies utilizing higher ERβ agonist doses, testing multiple times throughout the study, and/or testing during the dark-phase may prove useful in demonstrating these effects.

### 4.2. Effects on anxiety-and depression-like behaviors

Previous research has repeatedly demonstrated an important role for ERβ activation in reducing anxiety-and depression-like behaviors in OVX mice and rats (Frye and Walf, 2004; Lagunas et al., 2010; Li et al., 2014; Lund et al., 2005; Oyola et al., 2012; Rocha et al., 2005; Walf et al., 2004, Walf and Frye, 2005a, 2007). Surprisingly, the present study showed only an effect of E_2_ treatment on time in the center of and time spent rearing in the OF, but no other effects of any treatment in the OF, EPM, TST, or FST compared to vehicle. This discrepency, especially given the lack of effects of either ERβ agonist, could be due to a number of factors. Previous studies have shown that sensitivity to estrogenic compounds in these tasks is highly dependent upon dose, injection schedule, solvent used, and administration route (as reviewed in Walf and Frye, 2006). As discussed previously, we selected doses of E_2_, DPN, and EGX358 which had previously been shown to enhance memory consolidation in the Morris water maze, OP, and OR tasks when administered acutely in OVX mice (Gresack and Frick, 2006a; Hanson et al., 2018). Although 0.2 mg/kg E_2_ should produce physiological levels of E_2_ when administered via i.p. injection (Akinci and Johnston, 1997; Gresack and Frick, 2006a, 2006b), we cannot be sure of these levels after oral gavage. Additionally, 0.05 mg/kg DPN and 0.5 mg/kg EGX358 are relatively low compared to other studies in which 0.1–1.0 mg/kg DPN produced anxiolytic and anti-depressive effects (Eid et al., 2020; Lund et al., 2005; Oyola et al., 2012). Thus, future studies will be necessary to determine whether higher doses of chronically administered EGX358 or DPN reduce anxiety-and depression-like behaviors in these tasks.

Other factors to take into consideration include stress, administration route, and duration of treatment. A recent study demonstrated that daily subcutaneous injections of E_2_ (0.04 mg/kg), DPN (0.1 mg/kg), or the commercially available ERα agonist PPT (0.1 mg/kg) for 47 days in OVX mice all reduce time spent immobile in the TST, but that chronic unpredictable stress reverses these effects, such that DPN and PPT both increase time spent immobile in this task (Eid et al., 2020). These data suggest that elevated stress may block or even reverse the effects of estrogenic compounds on measures of anxiety-and depression-like behaviors. Although gavage is a more effective method of ensuring accurate oral dosing than water-or food-based delivery, it is also more invasive and stressful than these other methods (Machholz et al., 2012). Therefore, the stress associated with gavage, combined with the relatively low doses used, may have prevented E_2_, DPN, and EGX358 from influencing affective measures. Finally, ours is one of the longest-term studies examining the effects of ERβ agonist treatment on anxiety-and depression-like behaviors in OVX mice. Previous work found that 3–7 days is optimal for detecting anxiolytic and anti-depressive effects of E_2_ in both OVX rats and mice (Bernardi et al., 1989; Koss et al., 2004; Li et al., 2014; Luine et al., 1998; Rocha et al., 2005), and most studies demonstrating anxiolytic and anti-depressive actions of DPN in OVX rodents have administered the compound acutely or for up to 7 days (Lund et al., 2005; Oyola et al., 2012; Walf et al., 2004; Walf and Frye, 2005a, 2006). Thus, long-term ERβ agonist treatment may be less effective than short-term treatment in mitigating affective behaviors in OVX rodents. Future studies examining putative influences of dose, route of administration, treatment duration, and stress effects will be necessary to more fully understand the extent to which long-term oral administration of estrogenic compounds influences affective behaviors. Importantly, however, none of our treatments *increased* anxiety-and depression-like behaviors in any of our tasks, suggesting that anxiety-or depression-like behaviors are not side effects of long-term E_2_, DPN, or EGX358 treatment.

### 4.3. Effects on memory

Our results demonstrating that long-term oral EGX358 administration in OVX mice promotes spatial and object recognition memory formation in the OP and OR tasks fits well with previous results from our lab and others showing memory-enhancing effects of acute post-training ERβ agonism (Boulware et al., 2013; Hanson et al., 2018; Jacome et al., 2010; Pereira et al., 2014; Said et al., 2018; Walf et al., 2008; Zhao et al., 2011). Whereas many previous studies have demonstrated that a single post-training delivery of DPN either systemically (Hanson et al., 2018; Jacome et al., 2010; Walf et al., 2008) or directly into the hippocampus (Boulware et al., 2013; Hanson et al., 2018; Pereira et al., 2014) enhances OP and OR memory consolidation in young OVX rats and mice, and that 2 days of pre-training systemic injections recapitulates these effects (Jacome et al., 2010), the current study is one of few to demonstrate that long-term administration of ERβ agonists enhance memory. Previously, Zhao et al. (2011) demonstrated that treating OVX mice with an ERβ-selective phytoestrogen diet for 9 months enhanced spatial working memory in the Y-maze, and Said et al. (2018) reported that diet-based DPN treatment (~3.0 mg/kg/day) for 22 months enhanced spatial memory in the Barnes maze in OVX mice. Thus, the fact that EGX358, and to a lesser extent DPN, enhanced memory in the present study is consistent with previous reports of long-term ERβ agonist treatment.

We previously showed that acute DPN or EGX358 treatment given immediately post-training enhances OP and OR memory consolidation in young OVX mice (Hanson et al., 2018). These effects were observed after bilateral dorsal hippocampal infusion (DPN: 10 pg; EGX358: 100 pg and 1 ng), or systemic administration via i.p. injection or oral gavage (DPN: 0.05 mg/kg; EGX358: 0.5 mg/kg or 5 mg/kg) (Hanson et al., 2018). The present study adds to these findings by showing that long-term oral treatment of either DPN or EGX358 via gavage enhances spatial and object recognition memory in OVX mice. Although DPN treatment did not significantly enhance OR consolidation, there was a trend for such an effect that may have been revealed with an increased sample size.

Although this study was designed to determine effects of long-term treatment on memory and other factors, it is important to note that our effects on memory could be due not only to long-term treatment, but also to acute effects of treatment given 1 h prior to training and testing. Given our study design, we cannot necessarily exclude acute effects in the improvements seen here. However, it is noteworthy that daily oral treatment with DPN or EGX358 enhanced memory in these tasks to a similar extent as our previous acute studies (Hanson et al., 2018). Namely, here, we demonstrate that long-term treatment with DPN or EGX358 promoted time spent with the moved object in OP, such that DPN treatment and EGX358 treatment resulted in 36.6% and 24.9% more time with the moved object than chance. Additionally, long-term treatment increased time with the novel object in the OR task, such that DPN treatment and EGX358 treatment resulted in 20.7% and 19.6% more time spent investigating the novel object than chance. These findings are similar to our previous report, showing that acute gavage treatment of DPN and EGX358 resulted in 29.2% and 31.5% more time with the moved object in the OP task, and 30.7% and 29.9% more time with the novel object in the OR task (Hanson et al., 2018). It is interesting that there appears to be a slightly weaker effect of long-term treatment compared to acute treatment, although this could be due to a number of factors, including treatment timing, stress of repeated gavage, or time of testing relative to OVX surgery (1–2 weeks post-surgery in the acute study (Hanson et al., 2018) as compared to ~10 weeks, here). Although we have not tested effects of acute treatment on anxiety-like, depression-like, or vasomotor symptoms, we would expect similarly slight differences between acute and long-term administration in each of these measurements. Despite the minor difference between effect sizes of acute and long-term treatments, our finding that long-term oral treatment with our highly selective ERβ agonist, EGX358, enhanced memory consolidation in both OP and OR further lends support for its continued development as an ERβ-selective HT for menopause-related memory dysfunction.

### 4.4. Effects on body weight and overall health

Finally, we also show here that daily treatment with E_2_ or ERβ agonists does not reduce weight gain following OVX or cause adverse health problems including premature death. Interestingly, none of our estrogenic treatments prevented weight gain over time compared to vehicle. As E_2_ treatment has previously been shown to reduce OVX-induced weight gain in both mice (Couse and Couse and Korach, 1999; Litwak et al., 2014) and rats (Hertrampf et al., 2006; Sibonga et al., 1998; Wegorzewska et al., 2008), it was surprising that E_2_-treated mice did not exhibit significantly lower body weights than vehicle-treated mice. This lack of effect may be at least partially mediated by our use of a standard diet instead of a high-fat diet, as supported by previous rat (Weigt et al., 2012; Yokota-Nakagi et al., 2020) and mouse studies (Litwak et al., 2014). It is not surprising, however, to find that neither DPN nor EGX358 reduced weight gain compared to vehicle, as previous studies report mixed results, such that either no effect of phytoestrogen diets or ERβ agonist treatment was observed in rats (Hertrampf et al., 2007, 2008; Weigt et al., 2012), positive effects were found only under high-fat diet conditions in wild-type compared to ERβ knockout mice (Foryst-Ludwig et al., 2008), or negative effects were shown under standard diet conditions in mice (Said et al., 2018). These findings suggest a stronger ERα-mediated protective role in body weight regulation.

One potential confound with our body weight measurements is that the phytoestrogen content (350–650 mg/kg) of our rodent chow might have obscured effects of long-term estrogen treatments on body mass. Long-term consumption of diets containing phytoestrogens at roughly the same concentrations as our diet alleviate weight gain in OVX rats (Kishida et al., 2008; Kurrat et al., 2015) and mice (Kim et al., 2006) compared to phytoestrogen-free diets (Kim et al., 2006; Kishida et al., 2008; Kurrat et al., 2015). However, the only dose to effectively reduce body mass in Kim et al. (2006) was much higher than our standard chow (1500 mg/kg versus 350–650 mg/kg, respectively), suggesting that the dose used here may not affect body mass in mice. It is also possible that the phytoestrogen content in our chow affected other measures in this study, as previous work has demonstrated that long-term consumption of dietary phytoestrogens at similar or lower concentrations than our chow may confound effects of exogenous E_2_ on anxiety-and depression-like behaviors (Russell et al., 2017) and may have anxiolytic and depression-reducing effects similar to E_2_ treatment (Kageyama et al., 2010; Rodríguez-Landa et al., 2017) in OVX rats. However, that long-term E_2_ treatment here improved anxiety-like behaviors, and E_2_, DPN, and EGX358 improved vasomotor and memory outcomes, suggests that OVX mice require higher phytoestrogen concentrations than rats to affect these outcomes, which is supported by Kim et al. (2006). Regardless, a follow-up study examining our administration doses in OVX mice fed phytoestrogen-free diets may be helpful in determining whether diet may have confounded results presented here.

That long-term treatment of EGX358 did not increase body weight or result in premature removal from this study due to health complications or apparent stress suggests little adverse effects on overall health. These data complement our previous findings that a single i.p. injection of EGX358 did not affect liver, heart, or kidney tissues and that EGX358 does not facilitate breast cancer cell proliferation or bind to other nuclear hormone receptors (Hanson et al., 2018). Collectively, these two studies suggest minimal negative effects on general health, although more rigorous testing must be conducted in the future to examine other aspects of overall health (e.g., liver, skeleton, and muscles).

### 4.5. Conclusions

In conclusion, long-term oral treatment of young OVX mice with a novel, highly selective ERβ agonist, EGX358, reduced drug-induced vasodilation and enhanced spatial and object recognition memory without adverse effects on anxiety-or depression-like behaviors, body weight, or overall health. These data expand upon our previous findings in which acute post-training treatment with multiple doses of EGX358 via hippocampal infusion, i.p. injection, or oral gavage enhanced spatial and object recognition memory consolidation in young OVX mice (Hanson et al., 2018). Here, we used the lowest effective oral gavage dose from our previous study (Hanson et al., 2018) and found that EGX358 not only enhances memory but also mitigates drug-induced vasodilation, which expands the potential indications for EGX358 drug development to now include treatment of hot flashes.

It should be noted that OVX in young rodents does not perfectly recapitulate the human menopausal transition. In particular, OVX causes a very abrupt loss of circulating hormones, including E_2_, due to the sudden removal of the ovaries, whereas menopause in humans results in a gradual loss of circulating hormones due to ovarian senescence. However, the OVX model is useful preclinically to assess effects of exogenous hormones in the absence of endogenous circulating hormones. Thus, this model is a suitable first step in determining the potential efficacy of EGX358. Future studies will explore the dose-response in more detail, determine whether higher doses of EGX358 might reduce preclinical indices of menopause-related anxiety and depression, and test efficacy of EGX358 in middle-aged females. Although aging female rodents do not undergo complete follicular loss or experience drops in gonadotrophin levels (Wise, 2000), they do experience increases in follicle-stimulating hormone, luteinizing hormone, and E_2_ prior to a complete cessation of hormonal cycling (Frick et al., 1999; Lefevre and McClintock, 1988; Nelson et al., 1995), similar to the menopausal transition in human women. Therefore, the assessment of behavioral and physiological responses to EGX358 in middle-aged female mice will be a valuable next step in the preclinical development of this compound. Additionally, future work will utilize brain tissue collected after the completion of this study to determine the molecular effects of long-term administration of EGX358 in ERβ-laden brain regions implicated in menopausal symptoms, such as the dorsal hippocampus. Altogether, these results show promise for the development of ERβ-driven HTs, and specifically for EGX358 as a potential therapeutic agent that safely and effectively reduces menopausal symptoms, thereby enhancing the quality of life for older women.

## Figures and Tables

**Table 1:** Treatment group means for measures of anxiety-like behavior.

Task	Measure	Vehicle	E_2_	DPN	EGX358

Open field	Sample size (n)	10	10	8	10
	Time in center (s)	***8.32*** ±***1.84***	***17.95*** ±1.43*	***8.00*** ±***1.27***	***6.99*** ±***0.66***
	Time in middle (s)	103.80 ±13.36	104.60 ±9.43	84.14 ±12.17	94.28 ±12.57
	Time in outer (s)	487.90 ±14.43	477.50 ±12.55	507.00 ±12.77	498.10 ±12.82
	Center entries	13.90 ±2.12	18.11 ±2.35	12.75 ±1.81	10.80 ±1.30
	Middle entries	64.44 ±1.97	72.20 ±6.57	61.38 ±7.28	52.40 ±2.90
	Outer entries	49.56 ±1.78	51.50 ±3.93	48.88 ±5.95	41.90 ±2.31
	Distance in center (m)	1.14 ±0.18	1.54 ±0.14	1.24 ±0.22	0.98 ±0.13
	Distance in middle (m)	11.64 ±0.58	12.16 ±1.03	10.68 ±1.38	9.46 ±0.59
	Distance in outer (m)	26.99 ±1.41	29.43 ±1.78	30.92 ±1.62	31.82 ±3.93
	Total distance (m)	40.69 ±2.51	44.34 ±2.95	42.84 ±2.82	42.26 ±3.91
	Center boli	0.00 ±0.00	0.00 ±0.00	0.00 ±0.00	0.00 ±0.00
	Middle boli	0.20 ±0.13	0.11 ±0.11	0.14 ±0.14	0.20 ±0.13
	Outer boli	0.90 ±0.38	0.22 ±0.15	0.63 ±0.26	0.44 ±0.18
	Total boli	0.78 ±0.32	0.33 ±0.24	1.00 ±0.38	0.89 ±0.31
	Bouts Barbering	7.22 ±0.80	5.70 ±1.15	6.00 ±0.93	5.22 ±0.70
	Time barbering (s)	***19.86*** ±***2.50***	***12.82*** ±***1.64***	***20.13*** ±***2.86***	***13.89*** ±***0.81***
	Bouts rearing	98.11 ±5.85	117.40 ±5.94	107.00 ±8.59	107.60 ±5.17
	Time rearing (s)	***108.60*** ±***5.73***	***145.20*** ±***6.79***^#^	***122.30*** ±***13.05***	***135.90*** ±***6.18***
Elevated plus maze	Sample size (n)	10	10	8	10
	Time in open arms (s)	12.75 ±3.28	20.59 ±5.66	9.26 ±2.94	11.68 ±3.91
	Time in closed arms (s)	510.40 ±9.62	468.80 ±9.51	498.70 ±7.55	497.20 ±8.19
	Time in center (s)	79.36 ±4.74	84.87 ±4.70	84.63 ±6.98	94.56 ±6.95
	Open arm entries	1.80 ±0.47	3.30 ±7.61	1.43 ±0.57	2.33 ±0.69
	Closed arm entries	23.80 ±1.07	24.60 ±1.44	27.38 ±1.31	28.67 ±1.92
	Center entries	25.30 ±1.45	27.60 ±1.72	28.88 ±1.27	30.22 ±1.89
	Total entries	50.90 ±2.78	55.50 ±3.26	58.38 ±2.54	61.22 ±3.84
	Open arms boli	0.00 ±0.00	0.00 ±0.00	0.00 ±0.00	0.00 ±0.00
	Closed arms boli	0.00 ±0.00	0.11 ±0.11	0.00 ±0.00	0.00 ±0.00
	Center boli	0.00 ±0.00	0.00 ±0.00	0.00 ±0.00	0.00 ±0.00
	Total boli	0.00 ±0.00	0.11 ±0.11	0.00 ±0.00	0.00 ±0.00
	Peeking bouts	18.56 ±1.41	19.11 ±1.17	19.38 ±1.32	21.56 ±1.77
	Peeking time (s)	38.43 ±3.14	42.69 ±3.12	40.88 ±4.31	45.79 ±2.63

Measures in which the main effects of treatment is significant are highlighted in bold and italics.

* *p<*0.05 relative to all other groups.

# *p* <0.05 relative to vehicle.

**Table 2:** Treatment group means for measures of depression-like behavior.

Task	Measure	Vehicle	E_2_	DPN	EGX358

Tail suspension test	Sample size (n)	10	10	8	10
	Time immobile (s)	212.00 ±5.58	181.00 ±12.91	199.30 ±6.92	181.90 ±11.10
	Latency to first immobility (s)	8.90 ±1.89	9.07 ±1.78	12.58 ±4.43	5.84 ±1.21
	Total boli	2.50 ±0.79	2.10 ±0.53	2.38 ±0.60	0.89 ±0.39
Forced swim test	Sample size (n)	10	10	8	10
	Time immobile (s)	85.33 ±10.45	98.03 ±17.38	82.88 ±10.86	91.31 ±14.55
	Latency to first immobility (s)	3.45 ±1.10	1.53 ±0.44	2.75 ±1.18	6.59 ±2.04
	Total boli	4.56 ±0.50	4.67 ±0.37	4.00 ±0.54	3.56 ±0.38
